# Prognostic Significance of C-Reactive Protein in Lenvatinib-Treated Unresectable Hepatocellular Carcinoma: A Multi-Institutional Study

**DOI:** 10.3390/cancers15225343

**Published:** 2023-11-09

**Authors:** Taiki Okumura, Takefumi Kimura, Takanobu Iwadare, Shun-ichi Wakabayashi, Hiroyuki Kobayashi, Yuki Yamashita, Ayumi Sugiura, Satoru Joshita, Naoyuki Fujimori, Hideo Kunimoto, Michiharu Komatsu, Hideki Fukushima, Hiromitsu Mori, Takeji Umemura

**Affiliations:** 1 Department of Medicine, Division of Gastroenterology and Hepatology, Shinshu University School of Medicine, Matsumoto 390-8621, Japan; taiki0960@outlook.jp (T.O.); 22hm104g@shinshu-u.ac.jp (T.I.); shun_1@me.com (S.-i.W.); h_kobayashi1980@yahoo.co.jp (H.K.); unagichazuke.sansho.3694@gmail.com (Y.Y.); tumemura@shinshu-u.ac.jp (T.U.); 2 Department of Advanced Endoscopic Therapy, Shinshu University School of Medicine, Matsumoto 390-8621, Japan; 3 Department of Health Promotion Medicine, Shinshu University School of Medicine, Matsumoto 390-8621, Japan; joshita@shinshu-u.ac.jp; 4 Department of Internal Medicine, Sato Hospital, Nakano 389-2102, Japan; a19860530@gmail.com; 5 Department of Internal Medicine, Yodakubo Hospital, Nagawa 386-0603, Japan; 6 Department of Gastroenterology, Shinshu Ueda Medical Center, Ueda 386-8610, Japan; naoyuki.fuji@gmail.com; 7 Department of Gastroenterology, Nagano Municipal Hospital, Nagano 381-0006, Japan; hideo_kunimoto@hospital.nagano.nagano.jp; 8 Department of Gastroenterology, Suwa Red Cross Hospital, Suwa 392-0027, Japan; komichi@shinshu-u.ac.jp; 9 Department of Gastroenterology, Saku Central Hospital Advanced Care Center, Saku 385-0051, Japan; fukushima.hideki@sakuhp.or.jp; 10 Department of Gastroenterology, Nagano Red Cross Hospital, Nagano 380-0928, Japan; morih@live.jp; 11 Consultation Center for Liver Diseases, Shinshu University Hospital, Matsumoto 390-8621, Japan

**Keywords:** hepatocellular carcinoma, lenvatinib, C-reactive protein

## Abstract

**Simple Summary:**

Serum C-reactive protein (CRP) is an established biomarker for acute inflammation and has been identified as a prognostic indicator for hepatocellular carcinoma (HCC). It is especially important to verify the utility of CRP in HCC patients treated with lenvatinib because the benefits of lenvatinib vary greatly depending on each case. We retrospectively analyzed 125 HCC patients who received lenvatinib treatment at six centers. The low-CRP (<0.5 mg/dL) group exhibited significantly longer overall survival (OS) than the high-CRP (0.5≥ mg/dL) group (22.9 vs. 7.8 months, *p* < 0.001). In addition, time-to-treatment failure (TTF) was significantly longer in the low-CRP group (8.5 vs. 4.4 months, *p* = 0.007). In conclusion, baseline serum CRP level was identified as a useful prognostic factor in HCC patients receiving lenvatinib treatment.

**Abstract:**

Background: Serum C-reactive protein (CRP) is an established biomarker for acute inflammation and has been identified as a prognostic indicator for hepatocellular carcinoma (HCC). However, the significance of the serum CRP level, specifically in HCC patients treated with lenvatinib, remains unclear. Methods: We retrospectively analyzed 125 HCC patients who received lenvatinib treatment at six centers. Clinical characteristics were assessed to identify clinical associations between serum CRP and HCC prognosis. Results: The median overall serum CRP level was 0.29 mg/dL. The cohort was divided into two groups: the low-CRP group with a serum CRP < 0.5 mg/dL and the high-CRP group with a serum CRP ≥ 0.5 mg/dL. The low-CRP group exhibited significantly longer overall survival (OS) than the high-CRP group (22.9 vs. 7.8 months, *p* < 0.001). No significant difference was observed for progression-free survival (PFS) between the high- and low-CRP groups (9.8 vs. 8.4 months, *p* = 0.411), while time-to-treatment failure (TTF) was significantly longer in the low-CRP group (8.5 vs. 4.4 months, *p* = 0.007). The discontinuation rate due to poor performance status was significantly higher in the high-CRP group (*p* < 0.001). Conclusion: A baseline serum CRP level exceeding 0.5 mg/dL was identified as an unfavorable prognostic factor in HCC patients receiving lenvatinib treatment.

## 1. Introduction

Hepatocellular carcinoma (HCC) is the most prevalent primary liver malignancy and ranks as the sixth most common cancer worldwide [1]. Radical treatment options for early-stage HCC include surgical resection, transcatheter arterial chemoembolization (TACE), and local ablation therapy [2]. Systemic therapies, such as oral tyrosine kinase inhibitors (TKIs) and immunotherapy, are typically employed for intermediate and advanced HCC [3,4]. In recent years, several TKIs, including sorafenib, lenvatinib, regorafenib, ramucirumab, and cabozantinib, have demonstrated efficacy against unresectable HCC [3,5,6,7,8]. Notably, lenvatinib exhibited a superior antitumor effect in the REFLECT trial [7]. However, some patients do not receive the full benefits of lenvatinib due to tumor progression and such adverse events (AEs) as decreased appetite, fatigue, hypertension, and thyroid function abnormalities [7]. Accordingly, it is crucial to identify the patients who are likely to respond to lenvatinib prior to initiating therapy.

Recent studies have shown significant associations between C-reactive protein (CRP) levels and the prognosis for various malignancies [9,10]. CRP is a well-established marker of systemic inflammation and has been linked to tumor aggressiveness, metastasis, and poor outcome in different cancer types. However, information on the clinical utility of CRP for forecasting prognosis in lenvatinib-treated HCC is scarce [11]. The present study investigated the impact of CRP levels on estimating prognosis in patients with unresectable HCC treated with lenvatinib using clinical data obtained from six medical institutions in Japan. By evaluating the association between the CRP level and patient outcomes, we sought to reveal valuable insights into the potential role of CRP as a prognostic biomarker in lenvatinib-treated HCC. Our findings could contribute to optimizing treatment strategies and improving patient selection for lenvatinib therapy in this challenging clinical scenario.

## 2. Materials and Methods

### 2.1. Patients

We retrospectively enrolled 125 patients with unresectable HCC treated with lenvatinib between April 2018 and December 2021 at among 6 medical institutions in Nagano, Japan (Shinshu University Hospital, Shinshu Ueda Medical Center, Nagano Red Cross Hospital, Suwa Red Cross Hospital, Nagano Municipal Hospital, and Saku Central Hospital Advanced Care Center). Relevant clinical data, including medical history, laboratory results, radiological findings, and prior treatments, were collected from patient medical records. The start of the follow-up period was defined as the initiation date of lenvatinib treatment. The end of the follow-up period was determined as either the date of the final visit or death. Treatment response was assessed using computed tomography (CT) or magnetic resonance imaging (MRI) scans performed 6–12 weeks after treatment initiation and subsequently at approximately 3-month intervals, following the Response Evaluation Criteria in Solid Tumors guidelines, version 1.1. The Eastern Cooperative Oncology Group (ECOG) performance status was applied to estimate the patients’ general condition, with (0) indicating fully active, (1) restricted in strenuous activity, (2) restricted in work activity but ambulatory and capable of self-care, (3) capable of limited self-care (4) completely disabled, and (5) dead [12].

### 2.2. Diagnosis and Treatment of HCC

HCC diagnosis was determined based on imaging characteristics, including arterial hypervascularity and venous or delayed-phase washout observed using contrast-enhanced dynamic CT and/or MRI. Tumor progression was evaluated using the Barcelona Clinic Liver Cancer (BCLC) staging system [13]. Liver function was assessed using Child–Pugh classification system [14] and the albumin–bilirubin (ALBI) grade [15]. The treatment strategy for HCC in each patient was determined based on the Japanese HCC practice guidelines [16].

### 2.3. Lenvatinib Treatment

Oral lenvatinib was prescribed to patients with unresectable HCC. Acute infections and chronic infections, including tuberculosis, were ruled out before lenvatinib was initiated. No patients had concomitant collagen disease. The initial dosage was determined primarily based on body weight following the manufacturer’s guidelines for lenvatinib administration. Patients weighing over 60 kg received an initial dose of 12 mg once daily, with those weighing under 60 kg receiving an initial dose of 8 mg once daily. In patients with a risk of poor performance status (PS) or potential AEs, a lower initial dose of 4 mg once daily was prescribed at the discretion of the attending physician and with the patient’s consent. The lenvatinib dosage was adjusted or temporarily halted if unacceptable AEs or clinical tumor progression were observed.

### 2.4. Adverse Events

AEs were assessed and categorized using the National Cancer Institute Common Terminology Criteria for Adverse Events, version 5.0 [17]. As instructed by the drug manufacturer’s guidelines, the lenvatinib dose was reduced or interrupted in the event of severe AEs having a grade of ≥ 3 [7,18].

### 2.5. Ethics

This study was conducted in compliance with the ethical principles outlined in the Declaration of Helsinki (revised in 2013 by Fortaleza) and the Ethical Guidelines for Medical Research Involving Human Subjects (partially revised on 28 February 2017). 

### 2.6. Statistical Analysis

Statistical analysis and data visualization were carried out using StatFlex software, version 7.0.11 (Artech Co., Ltd., Osaka, Japan). Continuous baseline data including CPR are assessed just prior to the initiation of lenvatinib, expressed as the median and interquartile range, and statistically evaluated by means of the Mann–Whitney U test. Cox regression analysis was performed to determine the factors associated with prognosis. Overall survival (OS) was defined as the time from introduction of lenvatinib to death. Progression-free survival (PFS) was defined as the time from introduction of lenvatinib until first evidence of disease progression or death. Time-to-treatment failure (TTF) was defined as the time from the initiation of lenvatinib treatment to its discontinuation. Reasons for prematurely discontinuing treatment could include cancer progression but also adverse events, patient choice, or death. OS, PFS, and TTF were evaluated via Kaplan–Meier analysis, and differences between the respective groups were assessed using the log–rank test. Categorical variables were presented as a frequency (percentage) and analyzed using the chi-square test. All statistical tests were two-sided and evaluated at the 0.05 level of significance.

## 3. Results

### 3.1. Patient Characteristics

Table 1 presents the clinical characteristics of the cohort. Median age was 74 years, and the majority of patients (75.2%) were male. Forty-three patients received an initial lenvatinib dose of 12 mg/day, 65 received 8 mg/day, and 17 started with 4 mg/day due to reasons of advanced age and poor PS. The Child–Pugh score was 5 points in 72 patients, 6 points in 41 patients, and 7 points or more in 12 patients. BCLC staging was A, B, C, and D in 3, 65, 56, and 1 patients, respectively. Regarding tumor response to lenvatinib treatment, the number of complete response (CR), partial response (PR), stable disease (SD), and progressive disease (PD) patients was 2 (1.6%), 35 (28.0%), 33 (26.4%), and 35 (28.0%), respectively. Twenty patients were not evaluated due to early treatment failure, such as rapid worsening of PS or hepatic capacity. The treatment lines indicate the order in which lenvatinib was used in the chemotherapy. The 1st, 2nd, 3rd, 4th, and 5th treatment lines were used for 93, 22, 6, 3, and 1 patients, respectively. The median overall CRP level was 0.29 mg/dL. We defined the high-CRP group as CRP ≥ 0.5 mg/dL and the low-CRP group as CRP < 0.5 mg/dL in reference to previous reports on other organ cancers [19,20] and considering the median CRP in this study. Compared with the low-CRP group (69 patients), the high-CRP group (56 patients) had significantly lower serum albumin levels and prothrombin activity, as well as higher levels of aspartate aminotransferase (AST), alanine aminotransaminase, platelet counts, alfa-fetoprotein (AFP), and des-gamma-carboxy prothrombin (DCP). Liver function as assessed using the Child–Pugh score (*p* = 0.007) and modified albumin–bilirubin (mALBI) grade (*p* < 0.001) was significantly impaired in the high-CRP group. No patients received other therapies, such as radiofrequency ablation or TACE, during lenvatinib treatment. The median follow-up was 11.6 (95% confidence interval [CI]: 5.5–25.8) months. There were 95 OS events, 69 PFS events, and 104 TTF events during the follow-up.

### 3.2. Factors Associated with OS in All Patients

The median OS in the 125 patients treated with lenvatinib was 13.2 months (95% CI: 10.2–17.2 months) in this study. Univariable analysis revealed several prognostic factors significantly associated with OS, which included the treatment line (hazard ratio [HR]: 1.39, 95% CI: 1.06–1.81, *p* = 0.016), PS (HR: 1.33, 95% CI: 1.08–1.63, *p* = 0.006), BCLC stage (HR: 1.65, 95% CI: 1.14–2.39, *p* = 0.008), total bilirubin (HR: 1.52, 95% CI: 1.26–1.84, *p* < 0.001), albumin (HR: 0.41, 95% CI: 0.28–0.61, *p* < 0.001), prothrombin (%) (HR: 0.98, 95% CI: 0.97–0.99, *p* = 0.003), and CRP (HR: 1.74, 95% CI: 1.39–2.19, *p* < 0.001) (Table 2). Multivariable analysis using these factors was performed next to determine the independent factors associated with OS. We observed that PS (HR: 1.35, 95% CI: 1.07–1.71, *p* = 0.011), total bilirubin (HR: 1.55, 95% CI: 1.25–1.91, *p* < 0.001), albumin (HR: 0.60, 95% CI: 0.39–0.92, *p* = 0.018), and CRP (HR: 1.63, 95% CI: 1.25–2.13, *p* < 0.001) were all significant independent factors associated with OS. We performed an additional multivariable analysis for OS using variables previously reported as poor prognostic factors for unresectable HCC [21,22], including sex, the serum AFP level, the treatment line, the mALBI grade, the age at treatment initiation, and the serum CRP level (Table 3). The results revealed the mALBI grade, the age, and the serum CRP level to be significant poor prognostic factors for OS.

**Figure 1:**
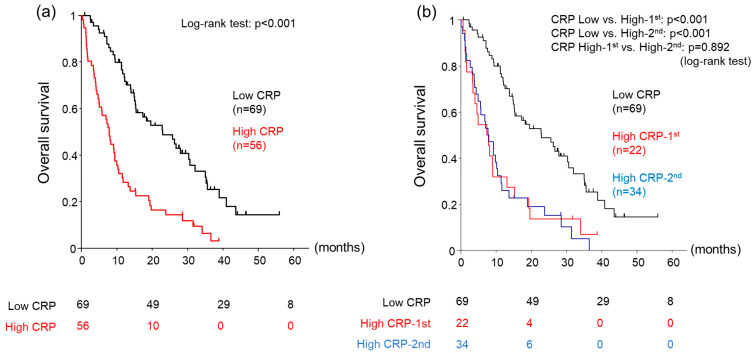
(**a**) Cumulative overall survival of patients divided into 2 groups using a cut-off value of 0.5 mg/dL serum C-reactive protein (CRP). (**b**) Cumulative overall survival of patients divided into 3 groups using cut-off values of 0.5 mg/dL and 1.0 mg/dL serum CRP. Low-CRP: CRP < 0.5 mg/d, High-CRP: CRP ≥ 0.5 mg/dL, High-CRP-^1st^: 0.5 ≤ CRP < 1.0 mg/dL, High-CRP-^2nd^: CRP ≥ 1.0 mg/dL.

We next divided the cohort into two groups based on a serum CRP cut-off level of 0.5 mg/dL (Table 1). The median OS was significantly longer in the low-CRP group than in the high-CRP group (22.9 vs. 7.8 months, log–rank test: *p* < 0.001) (Figure 1a). To identify a suitable cut-off value, we additionally divided the high-CRP group into the high-CRP 1st group (0.5 ≤ CRP < 1.0 mg/dL) and the high-CRP 2nd group (CRP ≥ 1.0 mg/dL) using a serum CRP of 1.0 mg/dL as the cut-off value (Table 4). OS was significantly longer in the low-CRP group (22.9 months) versus both the high-CRP 1st group (7.7 months, log–rank test: *p* < 0.001) and the high-CRP 2nd group (7.9 months, log–rank test: *p* < 0.001). OS was comparable between the high-CRP 1st and 2nd groups (log–rank test: *p* = 0.892) (Figure 1b).

### 3.3. Factors Associated with OS in Patients with ALBI Grade 1 and 2a

**Figure 2:**
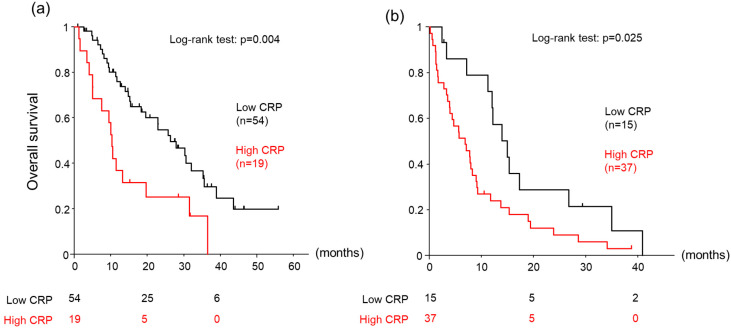
Cumulative overall survival of patients with a cut-off value of 0.5 mg/dL for serum C-reactive protein (CRP). (**a**) ALBI grades 1 and 2a, (**b**) ALBI grades 2b and 3. Low-CRP: CRP < 0.5 mg/dL, High-CRP: CRP≥ 0.5 mg/dL.

We next investigated factors associated with OS in HCC patients with preserved liver capacity, i.e., ALBI grades 1 and 2a. The median follow-up was 15.1 (8.7–28.5) months. Among the 73 patients in this subgroup, there were 47 OS events during the follow-up. Univariable analysis revealed significant associations with OS for age (HR: 1.04, 95% CI: 1.01–1.08, *p* = 0.007), AST (HR: 1.004, 95% CI: 1.000–1.008, *p* = 0.044), CRP (HR: 1.49, 95% CI: 1.06–2.11, *p* = 0.022), and DCP (HR: 1.001, 95% CI: 1.000–1.001, *p* = 0.010) (Table 5). In the subsequent multivariable analysis, age (HR: 1.05, 95% CI: 1.01–1.08, *p* = 0.006) and CRP (HR: 1.53, 95% CI: 1.08–2.16, *p* = 0.016) were confirmed as significant independent factors. The patients with an ALBI grade of 1 or 2a were separated into two groups based on a serum CRP level cut-off value of 0.5 mg/dL. OS was significantly longer in the low-CRP group than in the high-CRP group (26.3 vs. 10.2 months, log–rank test: *p* = 0.004) (Figure 2a).

### 3.4. Factors Associated with OS in Patients with ALBI Grades of 2b and 3

We next investigated factors associated with OS in HCC patients with poorer liver capacity, i.e., ALBI grades 2b and 3. The median follow-up was 7.9 (3.3–15.1) months. Among the 52 patients in this subgroup, there were 48 OS events during the follow-up. Univariable analysis revealed significant associations with OS for PS (HR: 1.49, 95% CI: 1.10–2.03, *p* = 0.011), total bilirubin (HR: 1.33, 95% CI: 1.07–1.64, *p* = 0.008), CRP (HR: 1.58, 95% CI: 1.11–2.26, *p* = 0.011), AST (HR: 1.005, 95%CI: 1.001–1.009, *p* = 0.017), and BCLC stage (HR: 1.63, 95% CI: 1.03–2.57, *p* = 0.036) (Table 6). In the subsequent multivariable analysis, performance status (HR: 1.44, 95% CI: 1.02–2.03, *p* = 0.036), total bilirubin (HR: 1.38, 95%CI: 1.09–1.75, *p* = 0.006), and CRP (HR: 1.78, 95% CI: 1.22–2.61, *p* = 0.003) were confirmed as significant independent factors. The patients with ALBI grades of 2b and 3 were separated into two groups based on a serum CRP level cut-off value of 0.5 mg/dL. OS was significantly longer in the low-CRP group than in the high-CRP group (14.9 vs. 6.9 months, log–rank test: *p* = 0.025) (Figure 2b).

### 3.5. Factors Associated with PFS in All Patients

In terms of prognostic factors associated with PFS in all patients treated with lenvatinib, no factors were significantly associated with PFS in the univariate analysis. PFS tended to be longer in the low-CRP group than in the high-CRP group, although this difference did not reach statistical significance (9.8 vs. 8.4 months, log–rank test: *p* = 0.411).

### 3.6. Factors Associated with TTF in All Patients

**Figure 3:**
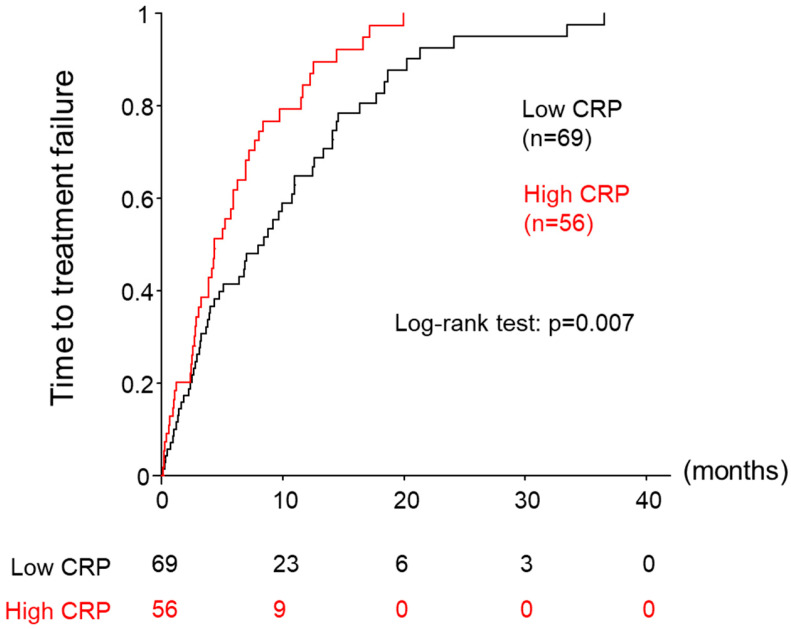
Cumulative incidence of treatment failure for patients divided into 2 groups using a cut-off value of 0.5 mg/dL for serum C-reactive protein (CRP). Low-CRP: CRP < 0.5 mg/dL, High-CRP: CRP ≥ 0.5 mg/dL.

Regarding prognostic factors associated with TTF in all patients treated with lenvatinib, univariable analysis identified age (HR: 1.030, 95% CI: 1.006–1.053, *p* = 0.012), hemoglobin (HR: 0.89, 95% CI: 0.81–0.98, *p* = 0.017), albumin (HR: 0.51, 95% CI: 0.36–0.74, *p* < 0.001), total bilirubin (HR: 1.32, 95% CI: 1.11–1.58, *p* = 0.002), AST (HR: 1.004, 95% CI 1.001–1.007, *p* = 0.021), prothrombin (%) (HR: 0.98, 95% CI 0.97–0.99, *p* = 0.004), and CRP (HR: 1.09, 95% CI 1.03–1.16, *p* = 0.006) as significantly associated with TTF (Table 7). In the subsequent multivariable analysis, age (HR: 1.03, 95% CI: 1.01–1.06, *p* = 0.008), albumin (HR: 0.55, 95% CI: 0.39–0.79, *p* = 0.001), and total bilirubin (HR: 1.28, 95% CI: 1.08–1.52, *p* = 0.004) were confirmed to be significant independent factors. At a CRP cut-off of≤ 0.5 mg/dL, TTF was significantly longer in the low-CRP group than in the high-CRP group (8.5 vs. 4.4 months, log–rank test: *p* = 0.007, Figure 3) but was not identified as an independent factor for TTF in multivariable analysis (Table 7).

### 3.7. Association between Reasons of Treatment Failure and CRP

**Figure 4:**
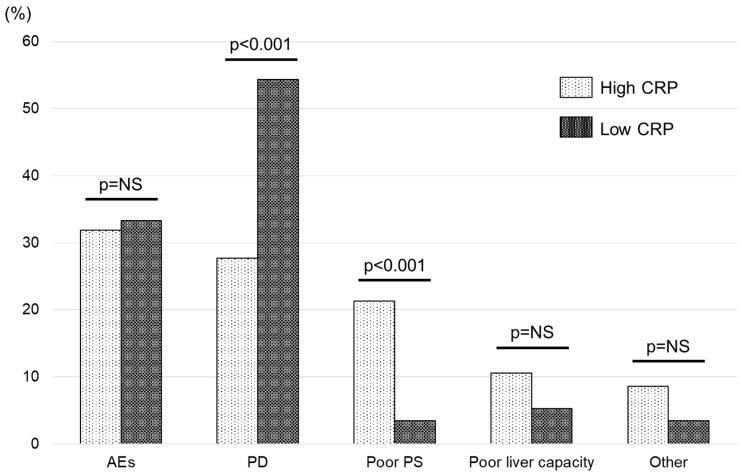
Comparison of reasons for treatment failure in all patients, divided into 2 groups using a cut-off value of 0.5 mg/dL for serum C-reactive protein (CRP). Low-CRP: CRP < 0.5 mg/dL, High-CRP: CRP ≥ 0.5 mg/dL. Abbreviations: AE, adverse event; PD, progressive disease; PS, performance status; NS, not significant.

The reasons for treatment failure in the high- and low-CRP groups are shown in Figure 4. In the high-CRP group, the number of patients who discontinued treatment due to AEs, PD, poor PS or liver capacity, or other reasons was 18 (31.9%), 15 (27.7%), 12 (21.3%), 6 (10.6%), and 5 (8.6%), respectively. In the low-CRP group, these values were 23 (33.3%), 36 (54.4%), 3 (3.5%), 4 (5.3%), and 3 (3.5%), respectively. Although the discontinuation rate due to AEs was comparable in both groups, the high-CRP group had a significantly higher rate due to poor PS (*p* < 0.001) and a significantly lower discontinuation rate due to PD (*p* < 0.001).

## 4. Discussion

A phase III clinical trial comparing lenvatinib and sorafenib in patients with HCC demonstrated its non-inferiority of lenvatinib in terms of OS and its superiority regarding antitumor efficacy [7]. However, it remains crucial to identify patients who can benefit from lenvatinib when considering treatment disruptions from anorexia, fatigue, and other possible AEs. In this study, we investigated the potential of the serum CRP level as a marker for estimating the prognosis of patients with unresectable HCC undergoing lenvatinib treatment. CRP is a widely used marker of systemic inflammation that has been associated with poor prognosis in various malignant tumors, including esophageal, colorectal, and pancreatic cancer [20,23,24]. Elevated CRP level has also been correlated to unfavorable outcomes in HCC patients receiving systemic anticancer therapy [11,21,25]. 

The current study demonstrated that the CRP level might serve as a predictor of OS in patients with unresectable HCC treated with lenvatinib. Our findings also indicated that a CRP cut-off value of 0.5 mg/dL could be more appropriate than the commonly used 1.0 mg/dL value. We observed no significant difference in prognosis between the high 1st group and the high 2nd group (Figure 1b), suggesting that a lower CRP cut-off value might be more effective in identifying poor prognosis groups, especially in Japanese patients and other Asian populations [21,26]. 

Apart from OS, we also investigated the relationship between serum CRP level and response rate, PFS, and TTF. While we found no significant association between CRP and response rate or PFS, we observed a significantly longer TTF in the low-CRP group (Figure 3). Furthermore, our analysis of treatment discontinuation revealed that patients in the low-CRP group were more likely to discontinue treatment due to disease progression, whereas those in the high-CRP group were more likely to discontinue due to PS (Figure 4). The high number of HCC patients with elevated CRP discontinuing treatment due to poor PS provides valuable information on appropriate drug selection.

Lastly, CRP is an acute-phase reactant synthesized by hepatocytes and associated with proinflammatory cytokines, particularly interleukin-6 (IL-6). Elevated CRP levels in patients with malignant tumors and poor prognosis have been attributed to various mechanisms. Our study, along with others, supports the conclusion that elevated CRP is associated with such prognostic factors as tumor burden and the BCLC stage. Tumor growth may induce tissue inflammation, thereby increasing CRP. In addition, the high expression of IL-6 and CRP may indicate changes in the cancer immune microenvironment. Several studies have reported that poorly differentiated cancers show higher tissue CRP expression and higher serum CRP levels [27,28]. Moreover, mesenchymal-like tumor cells and stromal cells common to the tumor microenvironment have also been shown to secrete cytokines, including IL-6, which is involved in the synthesis of CRP as well as its secretion [29] and significantly enhances the success of tumor growth and metastatic implantation in vivo [30,31]. Based on these studies, it has been indicated that progressing tumors enhance their development and metastasis, suggesting that cancer cell-induced inflammation promotes tumor progression and thus disease severity. In recent research on HCC, high IL-6 levels have been associated with the decreased secretion of interferon-γ and tumor necrosis factor-α from CD8+ T cells, as well as suppressed cytokine production and CD8+ T cell proliferation. This non-T cell inflammatory immunosuppressive tumor microenvironment may reduce the efficacy of anticancer drugs [32]. It is noteworthy that a high-CRP level has been reported to be a poor prognostic factor in HCC patients treated with immune checkpoint inhibitors [21,26]. Thus, a combination of factors likely contributes to elevated CRP levels in patients with malignancies and poor prognosis.

While the present study offers valuable insights, it is important to acknowledge its inherent limitations. First, our investigation relied on a retrospective observational design, which limited our ability to establish causal relationships. Second, due to certain constraints, we were unable to explore the correlation between CRP levels and the relative dose intensity of lenvatinib. Lastly, the evaluation of prognosis relative to medications other than lenvatinib was beyond the scope of our study.

## 5. Conclusions

In summary, this investigation revealed a significant association between baseline CRP levels and prognosis in patients with HCC undergoing lenvatinib treatment. Elevated CRP was identified as a substantial negative predictor of OS, even in patients with relatively healthier liver function. Importantly, a serum CRP level exceeding 0.5 mg/dL at baseline was determined to be an unfavorable prognostic factor in HCC patients treated with lenvatinib. Further research is warranted to validate our findings and explore the underlying mechanisms by which CRP influences treatment outcome in HCC.

## Figures and Tables

**Table 1:** Patient demographic features.

	All Patients (n = 125)	High-CRP CRP ≥ 0.5 mg/dL (n = 56)	Low-CRP CRP < 0.5 mg/dL (n = 69)	*p*-Value *
	Median (IQR)			
Age (years)	74 (69–78)	75 (70–77)	73 (69–80)	0.907
Male, n (%)	94 (75.2)	47 (83.9)	47 (68.1)	0.191
ECOG-PS, 0/1/2, n	99/18/8	37/12/7	62/6/1	0.151
Etiology, HCV/HBV/other, n	41/13/71	11/4/41	30/9/30	0.108
Total bilirubin (mg/dL)	0.78 (0.60–1.07)	0.80 (0.62–1.19)	0.70 (0.59–0.99)	0.078
Albumin (g/dL)	3.6 (3.2–4.0)	3.3 (3.0–3.7)	3.8 (3.5–4.1)	<0.001
AST, U/L	44 (30–63)	54 (39–78)	35 (25–46)	<0.001
ALT, U/L	29 (18–48)	32 (22–56)	26 (17–36)	0.039
Platelets, ×10^4^/μL	14.5 (10.4–21.1)	17.4 (11.5–25.6)	12.5 (10.1–17.4)	0.001
Prothrombin, (%)	88 (78–97)	85 (77–91)	92 (81–101)	0.006
CRP (mg/dL)	0.29 (0.10–1.11)	1.45 (0.81–3.42)	0.12 (0.06–0.23)	<0.001
AFP (ng/mL)	57.8 (5.4–868.5)	177.5 (6.3–1981.5)	12.0 (4.7–234.1)	0.019
DCP (mAU/mL)	182.0 (36.5–2559.8)	589.7 (110.2–15462.7)	85.0 (23.0–323.0)	<0.001
Child–Pugh score, 5/6/≥7, n	72/41/12	19/25/12	53/16/0	0.007
ALBI grade	−2.35 (−2.63 to −1.97)	−2.05 (−2.45 to −1.74)	−2.47 (−2.78 to −2.30)	<0.001
mALBI grade, 1/2a/2b/3, n	37/36/45/7	9/10/30/7	28/26/15/0	<0.001
BCLC stage, A/B/C/D, n	3/65/56/1	1/23/31/1	2/42/25/0	0.101
Initial dose, 4 mg/8 mg/12 mg, n	17/65/43	9/26/21	8/39/22	0.053
Treatment line, 1st/2nd/3rd/4th/5th	93/22/6/3/1	39/12/2/2/1	54/10/4/1/0	0.128
Follow-up duration (months)	11.6 (5.5–25.8)	7.8 (3.5–13.5)	15.4 (9.5–28.7)	<0.001
Treatment response, CR/PR/SD/PD/NE, n	2/35/33/35/20	0/16/14/13/13	2/19/19/22/7	0.136

* High-CRP (CRP ≥ 0.5 mg/dL) group vs. Low-CRP (CRP < 0.5 mg/dL) group. Abbreviations: CRP, C-reactive protein; IQR, interquartile range; ECOG-PS, Eastern Cooperative Oncology Group-Performance Status; HCV, hepatitis C virus; HBV, hepatitis B virus; AST, aspartate transaminase; ALT, alanine transaminase; AFP, α-fetoprotein; DCP, des-gamma-carboxy prothrombin; ALBI grade, albumin–bilirubin grade; mALBI grade, modified albumin–bilirubin grade; BCLC stage, Barcelona Clinic Liver Cancer stage; CR, complete response; PR, partial response; SD, stable disease; PD, progression disease; NE, not evaluated.

**Table 2:** Univariable and multivariable Cox regression analyses of prognostic factors for overall survival in HCC patients treated with lenvatinib (all patients).

	Univariable		Multivariable	
	HR (95% CI)	*p*-Value	HR (95% CI)	*p*-Value
Treatment line	1.39 (1.06–1.81)	0.016		
Performance status	1.33 (1.08–1.63)	0.006	1.35 (1.07–1.71)	0.011
BCLC stage	1.65 (1.14–2.39)	0.008		
Total bilirubin, mg/dL	1.52 (1.26–1.84)	<0.001	1.55 (1.25–1.91)	<0.001
Albumin, g/dL	0.41 (0.28–0.61)	<0.001	0.60 (0.39–0.92)	0.018
Prothrombin, %	0.98 (0.97–0.99)	0.003		
CRP, mg/dL	1.74 (1.39–2.19)	<0.001	1.63 (1.25–2.13)	<0.001

Abbreviations: HCC, hepatocellular carcinoma; HR, hazard ratio; 95% CI, 95% confidence interval; BCLC stage, Barcelona Clinic Liver Cancer stage; CRP, C-reactive protein.

**Table 3:** Multivariable Cox regression analysis for overall survival using variables previously reported as poor prognostic factors in HCC patients treated with lenvatinib (all patients).

	Multivariable	
	HR (95% CI)	*p*-Value
Male	1.17 (0.69–1.96)	0.561
AFP, ng/mL	1.00 (0.99–1.01)	0.257
Treatment line	1.29 (0.99–1.70)	0.062
mALBI grade	1.53 (1.17–1.99)	0.002
Age	1.05 (1.02–1.08)	<0.001
CRP, mg/dL	2.21 (1.38–3.55)	<0.001

Abbreviations: HCC, hepatocellular carcinoma; HR, hazard ratio; 95% CI, 95% confidence interval; AFP, α-fetoprotein; mALBI grade, modified albumin–bilirubin grade; CRP, C-reactive protein.

**Table 4:** Demographic features of patients in the high-CRP group.

	High-CRP (n = 56) CRP ≥ 0.5 mg/dL	High-CRP-^1st^ (n = 22) 0.5≤ CRP < 1.0 mg/dL	High-CRP-^2nd^ (n = 34) CRP ≥ 1.0 mg/dL	* *p*-Value
	Median (IQR)			
Age (years)	75 (70–77)	72 (64–77)	76 (70–78)	0.121
Male, n (%)	47 (83.9)	19 (86.4)	28 (82.4)	0.689
ECOG-PS, 0/1/2, n	37/12/7	16/2/4	21/10/3	0.153
Etiology, HCV/HBV/other, n (%)	11/4/41	4/1/17	7/3/24	0.793
Total bilirubin (mg/dL)	0.80 (0.62–1.19)	0.9 (0.8–1.2)	0.7 (0.6–1.1)	0.067
Albumin (g/dL)	3.3 (3.0–3.7)	3.4 (3.1–3.8)	3.3 (2.9–3.6)	0.274
AST, U/L	54 (39–78)	51 (30–71)	56 (40–103)	0.182
ALT, U/L	32 (22–56)	28 (21–42)	38 (23–64)	0.161
Platelets, ×10^4^/μL	17.4 (11.5–25.6)	13.0 (10.2–16.8)	21.8 (14.5–30.5)	0.003
Prothrombin, (%)	85 (77–91)	87 (83–95)	83 (76–87)	0.122
CRP (mg/dL)	1.45 (0.81–3.42)	0.71 (0.64–0.82)	2.81 (1.55–4.76)	<0.001
AFP (ng/mL)	177.5 (6.3–1981.5)	165.6 (6.0–1878.0)	177.5 (6.5–5466.8)	0.973
DCP (mAU/mL)	589.7 (110.2–15462.7)	161.0 (58.9–2559.8)	5374.0 (306.0–36246.0)	0.008
Child–Pugh score, 5/6/≥7, n	19/25/12	19/2/1	0/23/11	<0.001
ALBI grade	−2.05 (−2.45 to −1.74)	−2.08 (−2.48 to −1.88)	−2.05 (−2.43 to −1.45)	0.491
mALBI grade, 1/2a/2b/3, n	9/10/30/7	4/4/13/1	5/6/17/6	0.543
BCLC stage, A/B/C/D, n	1/23/31/1	1/11/9/1	0/12/22/0	0.161
Initial dose, 4 mg/8 mg/12 mg, n	9/26/21	2/10/10	7/16/11	0.424
Treatment line, 1st/2nd/3rd/4th/5th	39/12/2/2/1	17/3/0/2/0	22/9/2/0/1	0.173
Treatment response, CR/PR/SD/PD/NE, n	0/16/14/13/13	0/6/5/7/4	0/10/9/6/9	0.801

* High CRP-^1st^ (0.5 ≤ CRP < 1.0 mg/dL) group vs. High CRP-^2nd^ (CRP ≥ 1.0 mg/dL) group. Abbreviations: CRP, C-reactive protein; IQR, interquartile range; ECOG-PS, Eastern Cooperative Oncology Group-Performance Status; HCV, hepatitis C virus; HBV, hepatitis B virus; AST, aspartate transaminase; ALT, alanine transaminase; AFP, α-fetoprotein; DCP, des-gamma-carboxy prothrombin; ALBI grade, albumin–bilirubin grade; mALBI grade, modified albumin–bilirubin grade; BCLC stage, Barcelona Clinic Liver Cancer stage; CR, complete response; PR, partial response; SD, stable disease; PD, progression disease; NE, not evaluated.

**Table 5:** Univariable and multivariable Cox regression analyses of prognostic factors for overall survival in HCC patients treated with lenvatinib (ALBI grade 1 and 2a).

	Univariable		Multivariable	
	HR (95% CI)	*p*-Value	HR (95% CI)	*p*-Value
Age	1.04 (1.01–1.08)	0.007	1.05 (1.01–1.08)	0.006
AST, U/mL	1.004 (1.000–1.008)	0.044		
CRP, mg/dL	1.49 (1.06–2.11)	0.022	1.53 (1.08–2.16)	0.016
DCP, mAU/mL	1.001 (1.000–1.001)	0.010		

Abbreviations: HCC, hepatocellular carcinoma; ALBI grade, albumin bilirubin grade; HR, hazard ratio; 95% CI, 95% confidence interval; AST, aspartate transaminase; CRP, C-reactive protein; DCP, des-gamma-carboxy prothrombin.

**Table 6:** Univariable and multivariable Cox regression analyses of prognostic factors for overall survival in HCC patients treated with lenvatinib (ALBI grade 2b and 3).

	Univariable		Multivariable	
	HR (95% CI)	*p*-Value	HR (95% CI)	*p*-Value
Performance status	1.49 (1.10–2.03)	0.011	1.44 (1.02–2.03)	0.036
Total bilirubin, mg/dL	1.33 (1.07–1.64)	0.008	1.38 (1.09–1.75)	0.006
CRP, mg/dL	1.58 (1.11–2.26)	0.011	1.78 (1.22–2.61)	0.003
AST, U/mL	1.005 (1.001–1.009)	0.017		
BCLC stage	1.63 (1.03–2.57)	0.036		

Abbreviations: HCC, hepatocellular carcinoma; ALBI grade, albumin bilirubin grade; HR, hazard ratio; 95% CI, 95% confidence interval; AST, aspartate transaminase; CRP, C-reactive protein; BCLC, Barcelona Clinic Liver Cancer.

**Table 7:** Univariable and multivariable Cox regression analyses of prognostic factors for time-to-treatment failure in HCC patients treated with lenvatinib (all patients).

	Univariable		Multivariable	
	HR (95% CI)	*p*-Value	HR (95% CI)	*p*-Value
Age	1.030 (1.006–1.053)	0.012	1.03 (1.01–1.06)	0.008
Hemoglobin, g/dL	0.89 (0.81–0.98)	0.017		
Albumin, g/dL	0.51 (0.36–0.74)	<0.001	0.55 (0.39–0.79)	0.001
Total bilirubin, mg/dL	1.32 (1.11–1.58)	0.002	1.28 (1.08–1.52)	0.004
AST, U/L	1.004 (1.001–1.007)	0.021		
Prothrombin, %	0.98 (0.97–0.99)	0.004		
CRP, mg/dL	1.09 (1.03–1.16)	0.006		

Abbreviations: HCC, hepatocellular carcinoma; HR, hazard ratio; 95% CI, 95% confidence interval; AST, aspartate transaminase; CRP, C-reactive protein.

## Data Availability

The datasets used and/or analyzed in the current study are available from the corresponding author upon reasonable request.
